# Opioids for Osteoarthritis: Cross-Sectional Survey of Patient Perspectives and Satisfaction

**DOI:** 10.3390/jcm12072733

**Published:** 2023-04-06

**Authors:** Thomas J. Schnitzer, Rebecca L. Robinson, Lars Viktrup, Joseph C. Cappelleri, Andrew G. Bushmakin, Leslie Tive, Mia Berry, Chloe Walker, James Jackson

**Affiliations:** 1 Feinberg School of Medicine, Northwestern University, Chicago, IL 60611, USA; 2 Value, Evidence and Outcomes, Eli Lilly and Company, Indianapolis, IN 46285, USA; 3 Neuroscience, Eli Lilly and Company, Indianapolis, IN 46285, USA; 4 Statistical Research and Data Science Center, Pfizer Inc., New York, NY 10017, USA; 5 Internal Medicine, Global Medical Affairs, Pfizer Inc., New York, NY 10017, USA; 6 Real World Research, Adelphi Real World, Bollington SK10 5JB, UK

**Keywords:** opioid, osteoarthritis, prescription analgesic, real-world clinical practice, tramadol

## Abstract

Patients often take opioids to relieve osteoarthritis (OA) pain despite limited benefits and potential harms. This study aimed to compare cross-sectional perspectives of patients that were taking prescription opioid (N = 471) or nonopioid medications (N = 185) for OA in terms of satisfaction, expectations of effectiveness, and concerns. Patients prescribed opioids (>7 days) reported more prior treatments (2.47 vs. 1.74), greater mean pain intensity (5.47 vs. 4.11), and worse quality of life (EQ-5D-5L index value mean 0.45 vs. 0.71) than patients prescribed nonopioid medications (all *p* < 0.0001). Based on linear regression models adjusting for demographics and pain intensity, patients prescribed opioids were less satisfied with overall regimen (3.40 vs. 3.67, *p* = 0.0322), had less belief that medications were meeting effectiveness expectations (2.72 vs. 3.13, *p* < 0.0001), and had more concerns about treatments being “not very good” (3.66 vs. 3.22, *p* = 0.0026) and addiction (3.30 vs. 2.65, *p* < 0.0001) than patients prescribed nonopioid regimens. When the models were replicated for subgroups with ≥30 days’ medication regimen duration, the findings were consistent with the main analyses. Patients have concerns about the risk of opioid addiction, but those with greater disease burden and more prior treatments continue taking opioid regimens.

## 1. Introduction

The risks of addiction, misuse, and mortality associated with opioid use are well established [1,2,3]. Pain is the hallmark symptom of osteoarthritis (OA), and can be progressive in nature requiring personalized, dynamic, and long-term treatment. For chronic pain due to OA, opioids are known to be minimally effective and associated with safety and tolerability issues [4,5,6,7,8]. Treatment guidelines have moved away from the use of opioids for OA, including using them only as a last resort medication [9,10,11,12], to be prescribed at the lowest dose, and for the shortest duration [12]. Clinical guidelines for the management of OA consider the patients’ affected joint(s), comorbidity profile, and personal situation [9,10,11,12]. The American College of Rheumatology (ACR) guidelines for the nonsurgical management of hip and knee OA strongly recommended nonpharmacologic therapies including aerobic/resistance land-based or water-based exercise, and weight loss as appropriate. Nonpharmacologic therapies should be conducted alongside the receipt of medications, if required, including acetaminophen (for those without comorbidities), nonsteroidal anti-inflammatory drugs (NSAIDs for those without comorbidities; preferably topical formulations for knee OA), and intra-articular corticosteroids. Other nonpharmacologic and pharmacologic approaches also are recommended.

Despite these recommendations, both tramadol and non-tramadol opioids are prescribed for patients with OA in the United States [13,14,15] and elsewhere [16,17,18,19,20]. In the United States, opioid use for OA was stable during the period 2007–2014 [14,15]. In 2016, guidance to help primary care physicians communicate the risks and benefits of opioids to patients with chronic noncancer pain was published by the Centers for Disease Control and Prevention (CDC) [21]. This may have contributed to subsequently reported reductions in the prescribing of opioids for OA by primary care physicians, rheumatologists, and orthopaedic surgeons; the majority of these physicians were concerned about drug dependence [22].

Patients with OA also have concerns about medication addiction, and this may affect quality of life [23]. Generally, satisfaction with OA medications has been associated with expected changes in pain intensity levels [24,25,26]; however, there are few data reported that are specific to patients with OA taking opioids. Satisfaction with non-tramadol opioids is lower in patients with OA with moderate-to-severe pain compared with mild pain [27], and opioid use has been associated with reduced satisfaction with the functional change afforded by medications [28].

To our knowledge, research findings have been reported from the health systems’ or healthcare provider’s perspective but not from the patients’ perspective. Given the contrasting evidence of benefit and continued use of opioids for OA pain, a greater understanding of the characteristics and perspectives of patients with OA who are prescribed opioids is warranted and could help physicians who are aiming to reduce opioid prescribing and improve treatment outcomes.

The aims of this study were to describe the characteristics of patients with OA currently prescribed medication regimens with and without opioids, and to assess patient satisfaction with, expectations of effectiveness of, and concerns about, these medication regimens.

## 2. Materials and Methods

This study was based on data from the Adelphi Disease Specific Programme (DSP) for OA, a point-in-time (cross-sectional) survey of US physicians and their patients conducted between February and May 2017. Adelphi DSPs are large, multinational studies of clinical practice using common methodology designed to capture robust, real-world data that reflect current demographics, clinical presentation, and treatment patterns [29] (Figure S1). The DSP for OA was performed in compliance with the US Health Insurance Portability and Accountability Act 1996, and the Western Institutional Review Board granted an ethical waiver as it was considered to pose minimal risk to participants.

### 2.1. Study Population

To capture real-world clinical practice behaviors, physicians identified from public lists of healthcare providers were screened by telephone and were eligible to participate if they typically treated at least 10 patients with OA each month. Participating physicians (81 primary care physicians, 35 rheumatologists and 37 orthopaedic surgeons) completed electronic patient record forms for their next nine consecutive patients (aged ≥ 18 years) currently diagnosed with OA (≥1 joint, any location) and, after providing written informed consent, those patients self-completed a questionnaire about their OA.

### 2.2. Medication Regimens

Medication regimens and duration of use were physician-reported, included any route of administration, and excluded over-the-counter (OTC) medications. Patient groups included either medication regimens with or without opioids. Opioids included tramadol and non-tramadol opioids, prescribed as monotherapy or in combination with or alongside other pain medications. Nonopioid regimens excluded opioids, and included nonsteroidal anti-inflammatory drugs (NSAIDs), corticosteroids (intra-articular or oral), viscosupplements, acetaminophen, capsaicin, glycosaminoglycans, and “other” medications. The minimum duration of all drugs within each current medication regimen was 7 days. For example, patients prescribed opioids for 1–6 days were excluded, regardless of the duration of any other drugs, since it was considered that this was insufficient experience of the regimen to gauge satisfaction and other outcomes.

### 2.3. Demographic, Clinical and Treatment Characteristics

Physicians reported the patient’s demographic (age, sex, ethnicity) and clinical characteristics (body weight and height, details of affected joints including number and location, and comorbidities and medications currently received for concomitant conditions). In addition to current medication regimens, physicians reported other treatments for OA including type of recommended nonpharmacologic treatments, prescribed OTC medications, and type and number of prior prescription medications. Physicians were asked “Have you tried an opioid dose-sparing approach to this patient’s osteoarthritis treatment?” (options: I am currently trying this approach for this patient; I have tried in the past for this patient but not currently; no but I will consider in the future for this patient; this is something I will never consider for this patient).

Physicians were asked “For each drug therapy the patient is currently prescribed for their osteoarthritis please record all reasons which influenced your choice in selecting the patient’s current drug(s)”, with multiple selection from a list of 31 attributes (encompassing efficacy, safety, quality of life, cost considerations, and convenience/acceptability) allowed. Physicians were asked “Please record any current issues the patient may be having with their current drug regimen”, and their responses (28 options) were categorized as no current issues, lack of efficacy, patient decision, drug interactions/comorbidities, adverse events/tolerability issues, worries about addiction, and cost/access issues.

Patients rated their pain intensity, on average, over the last week, scored on an 11-point numeric rating scale from 0 (no pain) to 10 (worst possible pain). Patients assessed their functional limitations due to OA using the Western Ontario and McMaster Universities Osteoarthritis Index (WOMAC) * [30] Physical Function subscale; each of the 17 questions being scored on 11-point numeric rating scales (from no difficulty to extreme difficulty, with higher score indicating worse functioning). The EQ-5D-5L was used to assess health status with higher index value indicating better health status [31]. *© 1996 Nicholas Bellamy. WOMAC^®^ is a registered trademark of Nicholas Bellamy (CDN, EU, USA).

### 2.4. Outcomes

Patient-reported satisfaction with, expectations of, and concerns about, medications for OA assessed using 5-point Likert scales.

To assess overall satisfaction with medications prescribed for OA, patients were asked “Which of the following options best describes your overall satisfaction with the prescribed medicine(s) for your osteoarthritis?”, with response options: very satisfied, somewhat satisfied, neither satisfied nor dissatisfied, somewhat dissatisfied, or very dissatisfied.

To assess satisfaction with 11 different attributes of their current medication regimen for OA, patients were asked “How satisfied are you with your prescribed medicine(s) in relation to the factors on the grid below?”. Response options included: extremely dissatisfied, somewhat dissatisfied, neither dissatisfied nor satisfied, somewhat satisfied, and extremely satisfied. Scores for the attributes were combined to form four categories including satisfaction with (1) pain relief (provides short-term pain relief, provides long-lasting pain relief, eases your pain quickly), (2) functional change (helps keep you mobile and active, allows you to return to your usual activities, helps maintain your independence), (3) tolerability (the side effects of the medicine), and (4) convenience of medications (has clear and simple instructions, is convenient to take in terms of fitting into your schedule, is easy to remember to take, the cost of my medicine).

To assess the expectations of effectiveness of medications for OA, patients were asked “How is your current medicine(s) meeting your level of expectation in relation to how effective it is for your OA?”, with response options including: it is a great deal more effective than I expected, it is more effective than I expected, it matches my expectations, it is less effective than I expected, it is much less effective than I expected.

Patient concerns about medications for OA were assessed by asking “Thinking generally about your osteoarthritis and using a scale of 1 to 5 (where 1 = complete disagreement, [3 = neither agree nor disagree], and 5 = complete agreement with the statement), please circle how strongly you agree with the following statements (circle one number on each line only)”. The statements included: “I feel that the current treatments available for osteoarthritis are not very good” and “I am concerned about becoming addicted to my medicine”.

### 2.5. Statistical Analyses

To ensure information was available for assessment of treatment, an oversample of one patient per physician who had tried at least three medications for their OA pain was included. Patients were excluded if the only joint affected was the back since this may be characteristic of a separate clinical entity rather than OA.

Pain intensity scores and functional limitation scores were each categorized as mild (0–3), moderate (4–6), or severe (7–10) for analysis.

Currently prescribed medication for OA was investigated according to nonopioid regimens and opioid regimens. Patient characteristics were described using measures of central tendency and compared between medication regimens using the Student’s *t* test (continuous variables) or the chi-square test (categorical variables). In the contingency table analysis with an expected cell count of less than five, Fisher’s exact test (for 2-by-2 tables) [32] or Fisher’s generalised exact test was used (for r-by-c tables, where r or c or both exceed 2) [33]. Patient satisfaction, expectations of effectiveness, and concerns were compared between medication regimens using linear regression, adjusted for age, sex, ethnicity, body mass index, and pain intensity. For ease of interpretation, outcomes were transformed as necessary so that higher scores were associated with greater satisfaction, higher expectation of effectiveness, and greater concern. The main analyses were for medication duration ≥ 7 days; subgroup analyses with a medication duration of ≥30 days applied to all drugs were conducted, using the same models.

Data were managed and analyzed using SPSS version 7.5 (SPSS Inc., Chicago, IL, USA) and Stata version 17.0 (StataCorp, College Station, TX, USA). *p* < 0.05 was considered statistically significant.

## 3. Results

**Figure 1:**
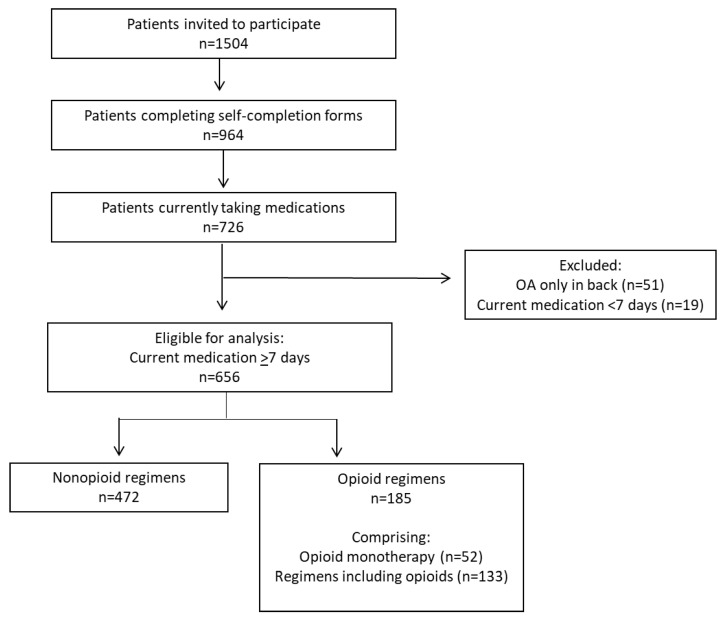
Patient disposition. To ensure information was available for assessment of treatment, an oversample of one patient per physician who had tried at least three medications for their OA pain was included. Abbreviation: OA, osteoarthritis.

Of 964 patients with OA who completed the DSP questionnaire, 656 were eligible for the current analyses including 471 patients in the nonopioid regimen group and 185 patients in the opioid regimen group (Figure 1).

Most patients in the nonopioid regimen (86.4%) and opioid regimen (93.5%) groups had been prescribed their medication regimen for at least 30 days (Table 1). For the nonopioid regimen group, physicians reported that most patients were prescribed an NSAID (84.9%) (Table 1); 77.5% of patients were prescribed one drug and 22.5% were prescribed ≥2 drugs; and 92.4% had never been prescribed an opioid for the treatment of OA. The average lines of OA medications were lower for nonopioid regimens vs. opioid regimens (1.74 vs. 2.47, respectively; *p* < 0.0001). Physicians reported that patients in the opioid regimens group were prescribed tramadol (56.2%) and/or non-tramadol opioids (50.3%) (Table 1), with 28.1% prescribed opioid monotherapy and 71.9% prescribed regimens including opioids (Figure 1). With respect to opioid dose sparing, physicians reported they were currently trying this for 19.8% of all patients and had tried it in the past for 7.8% of patients (Table 1). Nonpharmacologic therapies and OTC medications were widely recommended by physicians in both medication regimens (Table 1).

### 3.1. Characteristics of Patients across Medication Regimens

Patients in the nonopioid regimens group were younger and ethnicity was less diverse, but there were no differences in sex or the proportion who were obese, compared with patients in the opioid regimens group (Table 2). Patients in the nonopioid regimens group reported significantly lower pain intensity, fewer functional limitations, and better EQ-5D-5L index value compared with patients in the opioid regimens group (Table 2). Physicians reported that patients in the nonopioid regimens group had been diagnosed more recently, had fewer comorbidities, and had fewer joints affected with OA, compared with patients in the opioid regimens group (Table 2).

Across both medication regimens, physicians’ reasons for choice of the current medication were most often related to efficacy (58.5%) and safety (55.8%) (Table 3); physicians frequently selected “slows disease progression” (28.2%) with respect to efficacy and “good gastrointestinal safety” (25.0%) with respect to safety (Table S1). For most patients (78.8% for nonopioid regimens and 55.1% for opioid regimens), physicians reported there were no current issues with the medication regimen (Table 3); where there were issues, this was most frequently lack of efficacy (14.0% for nonopioid regimens and 28.1% for opioid regimens) (Table S2).

### 3.2. Patient-Reported Satisfaction with, Expectations of Effectiveness of, and Concerns about Medication Regimens (Patients Prescribed Current Medication for ≥7 Days)

For patients’ overall satisfaction with medication, satisfaction with attributes of medication (respect to pain relief, functional change, tolerability and convenience) and expectations of effectiveness of medication, least squares (LS) mean ratings for the nonopioid regimens group were significantly higher (greater satisfaction and met expectations better) than those for the opioid regimens group (Table 4).

For patients’ concerns about treatments being “not very good” and concerns about becoming addicted to medication, LS mean ratings for the nonopioid regimens group were significantly lower (indicating less concern) than those for the opioid regimens group (Table 4).

### 3.3. Subgroup of Patients Prescribed Current Medication for ≥30 Days

When the models were replicated for the subgroup of patients prescribed medication for ≥30 days, the results were consistent in terms of level of significance and directionality for satisfaction with, expectations of effectiveness of, and concerns about, medication regimens (Table 5) compared with the analyses for the overall population prescribed current medication for ≥7 days (Table 4).

## 4. Discussion

This study found differences in the clinical characteristics and perspectives of patients with OA currently prescribed opioids compared with those prescribed nonopioid regimens. Patients with OA who were prescribed opioids reported greater pain intensity than patients prescribed nonopioid medications.

After adjusting for demographics and pain intensity, patients with OA prescribed opioids were less satisfied with their medication, had less belief medications were meeting effectiveness expectations, and had more concerns about treatments being “not very good” and addiction than patients prescribed nonopioid regimens.

Compared with the nonopioid regimens group, the opioid regimens group in the current population had greater pain intensity, more joints affected, more functional limitations, more comorbidities including greater use of medications for these conditions, greater use of nonpharmacologic treatments for OA, and lower health status. Previous studies have reported greater levels of pain in patients with OA prescribed opioids compared with alternatives [27,34], and have also reported associations between opioid use and greater pain/disease burden and comorbidities [35,36,37]. Across various medications for OA, comorbidities have been associated with reduced health status [38]. There are few data comparing health status or quality of life for opioids vs nonopioids in patients with OA.

Pain has a key role in satisfaction [24,25,26,27], potentially complicating interpretation of findings across different medication regimens, underlining the importance of adjusting for pain severity in multivariate analyses. It is also likely that greater pain intensity could contribute to patients’ concerns about treatments being “not very good”. Such concerns were significantly less in patients prescribed nonopioids compared with opioids after adjusting for pain intensity. In line with the concerns of their physicians [22], the patients in the current study who were prescribed opioids reported they had concerns about addiction. It is possible that some patients’ concerns originated from discussions with their physician, especially considering the data that were collected after the publication of the CDC guideline [21] and the number of patients subject to a dose-sparing approach. Conversely, it is possible that some patients may not have been fully aware of the class of medication they were prescribed. Concerns about medication addiction have been linked to reduced quality life in patients with OA [23].

An earlier analysis based on the same patient population found that opioid use was associated with reduced satisfaction with the functional change afforded by medications [28]. The current analyses corroborated these findings, with significantly reduced satisfaction with functional change for the opioid regimens group compared with the nonopioid regimens group. For attributes of pain relief, tolerability afforded by medication and medication convenience, the opioid regimens group reported significantly lower satisfaction compared with the nonopioid regimens group.

To be eligible for the current analyses, patients had to be receiving their current medication regimen for at least 7 days. This relatively short minimum duration was to capture those patients with only a recent exposure to the medication, who might later find they were unable to tolerate it, resulting in discontinuation. The analyses were replicated based on a medication duration of at least 30 days to assess the perspectives of the subgroup who were stable on their medication regimen and considering that more than 7 days might be needed for evaluating the efficacy of some drugs. The results of the subgroup analyses (≥30 days’ medication regimen duration) were like the main analyses (≥7 days’ medication regimen duration), which reflects the fact that most patients in the study had been receiving their medication regimen for ≥30 days, with the average duration of use being approximately 9 months.

This study has some limitations. The multivariate analyses were adjusted for demographics and pain intensity; further analyses would be needed to determine what other factors might be associated with the observed differences. These findings may not be generalizable to the whole OA population, other specialties, or other countries. Many of the patients were prescribed more than one medication and various OTC medications and nonpharmacologic therapies, so their ability to differentiate the effects specific to their prescribed medication may be limited. Nonpharmacologic treatments are an important component in the overall treatment of OA [9,10,11,12,34,39,40]; yet, these analyses only address rates of satisfaction, expectations, or concerns with pharmacologic treatments. Further, although use of nonpharmacologic treatments were higher in the opioid regimen group, data on duration of these therapies were limited. Prior analysis from this patient population concluded that most patients are recommended or prescribed nonpharmacologic treatments alongside prescription medications with weight loss, exercise, and physical therapy being the most common [34].

## 5. Conclusions

This study showed that patients prescribed opioid regimens had greater pain intensity, more joints affected, more functional limitations, more comorbidities, greater use of nonpharmacologic treatments, and lower health status. After adjusting for demographics and pain intensity, patients with OA who were prescribed opioids were less satisfied, had less belief that medications were meeting effectiveness expectations, and had more concerns about treatments being “not very good” and addiction than patients prescribed nonopioid regimens. Though patients and their physicians have concerns about the effectiveness and risks of opioids, long-term use is common. 

## Figures and Tables

**Table 1:** Treatment characteristics of patients with OA (physician-reported).

	Nonopioid Regimens (n = 471)	Opioid Regimens (n = 185)	*p* Value
Currently prescribed medication, n (%)			
NSAID	400 (84.9)	103 (55.7)	<0.0001
Tramadol	0 (0.0)	104 (56.2)	NA
Non-tramadol opioid	0 (0.0)	93 (50.3)	NA
Corticosteroid	39 (8.3)	39 (21.1)	<0.0001
Viscosupplementation	27 (5.7)	9 (4.9)	0.6606
Other ^a^	122 (25.9)	68 (36.8)	0.0058
Current medication regimen duration, mean (SD), week ^b^	37.59 (32.13)	37.81 (33.28)	0.9364
Current medication regimen duration ≥30 days, n (%) ^b^	407 (86.4)	173 (93.5)	0.0105
Lines of OA medication, mean (SD)	1.74 (0.93)	2.47 (0.94)	<0.0001
Over-the-counter medication recommended, n (%)	147 (31.2)	70 (37.8)	0.1045
Nonpharmacologic therapy, n (%)			
Physical or occupational therapy	195 (41.4)	116 (62.7)	<0.0001
Acupuncture	25 (5.3)	19 (10.3)	0.0222
TENS	15 (3.2)	28 (15.1)	<0.0001
CBT / psychotherapy	7 (1.5)	11 (5.9)	0.0017
Opioid dose-sparing approach for this patient, n (%) ^c^			<0.0001
Currently trying	79 (16.8)	51 (27.6)	
Tried in the past but not currently	19 (4.0)	32 (17.3)	
No but will consider in the future	206 (43.7)	67 (36.2)	
Never will consider	167 (35.5)	35 (18.9)	

^a^ “Other” includes glycosaminoglycans (8.5%), nonopioid, and non-NSAID analgesics such as acetaminophen or capsaicin (20.7%) and other medications that were used in <5% of patients, including immunosuppressants, antidepressants, anticonvulsants, disease-modifying antirheumatic drugs, bisphosphonates, muscle relaxants, biologics, and vitamins/herbs. ^b^ Based on time since starting treatment (for patients receiving their first treatment) or time since last treatment regimen change (regardless of whether this was a change in drug or treatment addition/removal). If more than one medication was included in the current regimen, duration was calculated based on mean time across all current medications within the regimen. ^c^ “Opioid dose-sparing approach” was not defined during the survey. Physicians were asked: “Have you tried an opioid dose-sparing approach to this patient’s osteoarthritis treatment?”. Abbreviations: CBT, Cognitive behavior therapy; NSAID, nonsteroidal anti-inflammatory drug; OA, osteoarthritis; SD, standard deviation. TENS, transcutaneous electrical nerve stimulation; NA not applicable.

**Table 2:** Demographic and clinical characteristics of patients with OA.

	Nonopioid Regimens (n = 471)	Opioid Regimens (n = 185)	*p* Value
Age, y			
Mean (SD)	64.70 (11.18)	66.86 (11.32)	0.0268
<55, n (%)	81 (17.2)	27 (14.6)	0.0452
55–64, n (%)	143 (30.4)	48 (25.9)	
65–74, n (%)	162 (34.4)	63 (34.1)	
≥75, n (%)	85 (18.0)	47 (25.4)	
Female, n (%)	286 (60.7)	109 (58.9)	0.6712
Ethnicity, n (%)			0.0377
White/Caucasian	375 (79.6)	135 (73.0)	
African American	54 (11.5)	24 (13.0)	
Native American	2 (0.4)	1 (0.5)	
Asian-Indian subcontinent	5 (1.1)	1 (0.5)	
Asian—other	5 (1.1)	1 (0.5)	
Chinese	1 (0.2)	0 (0.0)	
Hispanic/Latino	28 (5.9)	17 (9.2)	
Middle Eastern	0 (0.0)	3 (1.6)	
Mixed race	1 (0.2)	3 (1.6)	
Body mass index, kg/m^2^			
Mean (SD)	29.09 (5.66)	29.98 (6.44)	0.0822
<30, n (%)	294 (62.4)	105 (56.8)	0.1815
≥30, n (%)	177 (37.6)	80 (43.2)	
Number of comorbidities, mean (SD) ^e^	2.13 (1.76)	3.79 (2.50)	<0.0001
Number of medications currently received to treat concomitant conditions, mean (SD)	2.08 (1.76)	3.76 (2.55)	<0.0001
Number of affected joints, mean (SD)	3.19 (2.44)	4.42 (2.68)	<0.0001
Years since OA diagnosis, mean (SD) ^a^	2.26 (2.91)	4.15 (4.17)	<0.0001
Location of affected joints, n (%)			
Knee	302 (64.1)	123 (66.5)	0.5678
Hip	162 (34.4)	78 (42.2)	0.0631
Back	146 (31.0)	99 (53.5)	<0.0001
Other	221 (46.9)	115 (62.2)	0.0004
Pain intensity, on average in the last week ^b^			
Mean score (SD)	4.11 (2.30)	5.47 (2.18)	<0.0001
Mild (0–3), n (%)	216 (46.7)	43 (23.9)	<0.0001
Moderate (4–6), n (%)	171 (36.9)	78 (43.3)	
Severe (7–10), n (%)	76 (16.4)	59 (32.8)	
WOMAC Physical Function ^c^			
Mean score (SD)	3.13 (2.11)	4.98 (2.48)	<0.0001
Mild (0–3), n (%)	294 (67.4)	50 (30.9)	<0.0001
Moderate (4–6), n (%)	101 (23.2)	61 (37.7)	
Severe (7–10), n (%)	41 (9.4)	51 (31.5)	
EQ-5D-5L index value, mean (SD) ^d^	0.71 (0.22)	0.45 (0.37)	<0.0001

^a^ Sample size: nonopioid regimens (n = 267), opioid regimens (n = 64). ^b^ Patient-reported. Sample size: nonopioid regimens (n = 463), opioid regimens (n = 180). ^c^ Patient-reported. Sample size: nonopioid regimens (n = 436), opioid regimens (n = 162). ^d^ Patient-reported. Sample size: nonopioid regimens (n = 458), opioid regimens (n = 176). ^e^ Most common comorbid conditions include: cardiological (66.2%), endocrine (48.0%), neurological/psychological (31.7%), respiratory conditions (9.0%), chronic low back pain (15.7%), other musculoskeletal or painful conditions (17.8%). Abbreviations: OA, osteoarthritis; SD, standard deviation; WOMAC, Western Ontario and McMaster Universities Osteoarthritis Index.

**Table 3:** Treatment considerations and issues of patients with OA (physician-reported).

	Nonopioid Regimens (n = 471)	Opioid Regimens (n = 185)
Reasons for choice of current medication ^a^		
Efficacy	261 (55.4)	123 (66.5)
Safety	267 (56.7)	99 (53.5)
Quality of life	88 (18.7)	58 (31.4)
Cost considerations	58 (12.3)	26 (14.1)
Convenience/acceptability	59 (12.5)	49 (26.5)
Issues with current medication regimen ^b^		
No current issues	371 (78.8)	102 (55.1)
Lack of efficacy	66 (14.0)	52 (28.1)
Patient decision	13 (2.8)	12 (6.5)
Drug interactions/comorbidities	2 (0.4)	5 (2.7)
Adverse events or tolerability issues	26 (5.5)	54 (29.2)
Worries about addiction	4 (0.8)	16 (8.6)
Cost or access issues	3 (0.6)	3 (1.6)

Data are n (%). ^a^ Physicians were asked “For each drug therapy the patient is currently prescribed for their osteoarthritis please record all reasons which influenced your choice in selecting the patient’s current drug(s)”. Data reflect an average of all drugs within the regimen if the patient was prescribed >1 drug. See Table S1 for details of all response options. ^b^ Physicians were asked “Please record any current issues the patient may be having with their current drug regimen”. See Table S2 for details of all response options.

**Table 4:** Patient-reported overall satisfaction with, expectations of effectiveness of, concerns about and satisfaction with attributes of medications for OA, for all patients treated for ≥7 days.

		Nonopioid Regimens		Opioid Regimens		Medication Regimen Comparison	
Models	Scores (1 to5) Higher Score =	n	LS Mean (95% CI)	n	LS Mean (95% CI)	LS Mean Difference (95% CI)	*p* Value
Satisfaction	Greater satisfaction						
overall, with regimen		392	3.67 (3.50, 3.84)	161	3.40 (3.16, 3.64)	−0.27 (−0.52, −0.02)	0.0322
with pain relief		417	3.52 (3.38, 3.67)	169	3.29 (3.10, 3.48)	−0.23 (−0.42, −0.05)	0.0132
with functional change		415	3.61 (3.47, 3.74)	168	3.22 (3.06, 3.38)	−0.39 (−0.54, −0.23)	<0.0001
with tolerability		407	3.63 (3.48, 3.78)	168	3.42 (3.27, 3.58)	−0.21 (−0.39, −0.03)	0.0208
with convenience		414	3.92 (3.80, 4.04)	168	3.64 (3.52, 3.76)	−0.28 (−0.42, −0.14)	<0.0001
Expectations of effectiveness of medications	Met expectation better	414	3.13 (2.98, 3.28)	167	2.72 (2.55, 2.90)	−0.41 (−0.59, −0.24)	<0.0001
Concerns	Stronger agreement						
about treatments not being very good		463	3.22 (3.01, 3.42)	180	3.66 (3.41, 3.92)	0.45 (0.16, 0.73)	0.0026
about becoming addicted		414	2.65 (2.41, 2.89)	165	3.30 (3.00, 3.59)	0.65 (0.37, 0.93)	<0.0001

Linear regression. Least squares mean adjusted for age, sex, race/ethnicity, body mass index, and pain intensity. Abbreviations: CI, confidence interval; LS, least squares; OA, osteoarthritis.

**Table 5:** Patient-reported overall satisfaction with expectations of effectiveness of, concerns about and satisfaction with attributes of, medications for OA, for all patients treated for ≥30 days.

		Nonopioid Regimens		Opioid Regimens		Medication Regimen Comparison	
Models	Scores (1 to5) Higher Score =	n	LS Mean (95% CI)	n	LS Mean (95% CI)	LS mean Difference (95% CI)	*p* Value
Satisfaction	Greater satisfaction						
overall, with regimen		346	3.68 (3.48, 3.87)	151	3.38 (3.12, 3.64)	−0.30 (−0.56, −0.03)	0.0267
with pain relief		364	3.54 (3.38, 3.70)	158	3.26 (3.07, 3.46)	−0.27 (−0.47, −0.08)	0.0056
with functional change		363	3.60 (3.45, 3.75)	157	3.18 (3.01, 3.34)	−0.42 (−0.59, −0.25)	<0.0001
with tolerability		355	3.61 (3.45, 3.76)	157	3.42 (3.26, 3.58)	−0.19 (−0.37, −0.00)	0.0469
with convenience		362	3.88 (3.76, 4.01)	157	3.63 (3.50, 3.75)	−0.26 (−0.40, −0.12)	0.0004
Expectations of effectiveness of medications	Met expectation better	362	3.10 (2.95, 3.25)	157	2.68 (2.50, 2.86)	−0.42 (−0.60, −0.24)	<0.0001
Concerns	Stronger agreement						
about treatments not being very good		402	3.27 (3.03, 3.50)	168	3.69 (3.43, 3.95)	0.42 (0.12, 0.73)	0.0074
about becoming addicted		355	2.61 (2.37, 2.86)	154	3.26 (2.96, 3.56)	0.65 (0.37, 0.92)	<0.0001

Linear regression. Least squares mean adjusted for age, sex, race/ethnicity, body mass index, and pain intensity. Abbreviations: CI, confidence interval; LS, least squares; OA, osteoarthritis.

## Data Availability

The data that support the findings of this study are available from Adelphi Real World, but restrictions apply to the availability of these data, which were used under license for the current study and so are not publicly available. However, data are available from the authors upon reasonable request and with permission from Adelphi Real World.

## Supplementary Materials

The following supporting information can be downloaded at: https://www.mdpi.com/article/10.3390/jcm12072733/s1, Table S1. Reasons for choice of current medication for patients with osteoarthritis (physician-reported); Table S2. Issues with current medication regimen of patients with osteoarthritis (physician-reported). Figure S1. Adelphi Disease Specific Programme Methodology.
