# Supplementary material for: Opioids for Osteoarthritis: Cross-Sectional Survey of Patient Perspectives and Satisfaction
Source: J Clin Med. 2023 Apr 6;12(7):2733. doi: 10.3390/jcm12072733 (PMC10095440; doi:10.3390/jcm12072733)

Journal of
Clinical Medicine

Supplementary Materials

Table S1 Reasons for choice of current medication for patients with osteoarthritis (physician-reported)

Table S2 Issues with current medication regimen of patients with osteoarthritis (physician-reported)

Figure S1. Adelphi Real World - Disease Specific Programmes (DSPs) Methodology

Table S1 Reasons for choice of current medication for patients with osteoarthritis (physician-reported)

	**Nonopioid regimens (n = 471)**	**Opioid regimens (n = 185)**
**Efficacy**		
Effective relief of pain at rest	39 (8.3)	24 (13.0)
Effective relief of pain on movement	61 (13.0)	43 (23.2)
Reduces number of tender/swollen joints	45 (9.6)	22 (11.9)
Maintains symptomatic relief over time without need to increase dose/alter frequency	33 (7.0)	31 (16.8)
Rapid onset of action	64 (13.6)	16 (8.6)
Effective in controlling acute flares	38 (8.1)	17 (9.2)
Slows disease progression	133 (28.2)	52 (28.1)
Delays the need for surgery	74 (15.7)	31 (16.8)
**Safety**		
Safe long-term use	104 (22.1)	27 (14.6)
Good gastrointestinal safety	138 (29.3)	26 (14.1)
Low incidence of serious adverse events/severe side effects	37 (7.9)	16 (8.6)
Suitable for patients with cardiovascular risk	61 (13.0)	10 (5.4)
Can be used continuously (without a drug break)	28 (5.9)	8 (4.3)
Appropriate for use in the elderly (>65 years)	50 (10.6)	21 (11.4)
No need for frequent monitoring/testing	44 (9.3)	17 (9.2)
Low risk of addiction/abuse	16 (3.4)	49 (26.5)
Can be used in combination with other pain drugs	16 (3.4)	11 (5.9)
**Quality of life**		
Improves/maintains attendance at work	16 (3.4)	10 (5.4)
Improves/maintains ability to perform activities of daily living/leisure activities	33 (7.0)	18 (9.7)
Improves/maintains relationships/social life	18 (3.8)	13 (7.0)
Improves patient functionality	53 (11.3)	25 (13.5)
Improves patient's sleep patterns/quality of sleep	16 (3.4)	10 (5.4)
Improves/maintains patient's independence	26 (5.5)	15 (8.1)
**Cost considerations**		
Low out of pocket cost/affordability for patient	58 (12.3)	26 (14.1)
Low cost/affordable for physician/practice	-	-
**Convenience/acceptability**		
Ease of administration for patient	17 (3.6)	14 (7.6)
Convenient dosing schedule	22 (4.7)	20 (10.8)
Physician familiarity/experience	9 (1.9)	6 (3.2)
On formulary/hospital approved drug list	10 (2.1)	7 (3.8)

Can be used in combination with other osteoarthritis therapies	11 (2.3)	11 (5.9)
Accordance with treatment guidelines	2 (0.4)	4 (2.2)

Data are n (%). All patients prescribed current medication regimen for ≥7 days. The data reflect responses for multiple drugs per patient if the patient was prescribed >1 drug. Physicians were asked, "For each drug therapy the patient is currently prescribed for their osteoarthritis please record all reasons which influenced your choice in selecting the patient's current drug(s)".

Table S2 Issues with current medication regimen of patients with osteoarthritis (physician-reported)

**Category**	**Response options**	**Nonopioid regimens (n = 471)**	**Opioid regimens (n = 185)**
No current issues	No current issues	371 (78.8)	102 (55.1)
Lack of efficacy	Lack of efficacy	66 (14.0)	52 (28.1)
Patient decision	Poor patient compliance	10 (2.1)	3 (1.6)
	Patient requested change	-	-
	Inconvenient dosing frequency	7 (1.5)	9 (4.9)
	Treatment break/holiday	-	-
Drug interactions/comorbidities	Drug interactions	2 (0.4)	2 (1.1)
	Comorbidities	0 (0.0)	3 (1.6)
Adverse events or tolerability issues	Tiredness/fatigue	11 (2.3)	27 (14.6)
	Constipation	4 (0.8)	28 (15.1)
	Nausea/vomiting	7 (1.5)	4 (2.2)
	Drowsiness	6 (1.3)	18 (9.7)
	Dizziness	1 (0.2)	9 (4.9)
	Difficulty concentrating	0 (0.0)	4 (2.2)
	Blurred vision	0 (0.0)	1 (0.5)
	Headaches	3 (0.6)	5 (2.7)
	Sleep problems/disturbances	0 (0.0)	6 (3.2)
	Dry mouth	5 (1.1)	14 (7.6)
	Skin irritation	-	-
	Administration reaction	1 (0.2)	0 (0.0)
Worries about addiction	Worries about addiction	4 (0.8)	16 (8.6)
Cost or access issues	Cheaper drug	1 (0.2)	1 (0.5)
	Insurance restrictions	3 (0.6)	1 (0.5)
	Patient out of pocket expense	0 (0.0)	2 (1.1)
	Formulary driven switch	-	-
	Fewer administrative hurdles	-	-
	Resource changes in clinic/practice forced switch	-	-
Other	Other (specify)	3 (0.6)	2 (1.1)

Data are n (%). All patients prescribed current medication regimen for ≥7 days. Physicians were asked “Please record any current issues the patient may be having with their current drug regimen”.

Figure S1. Adelphi Real World - Disease Specific Programmes (DSPs) Methodology

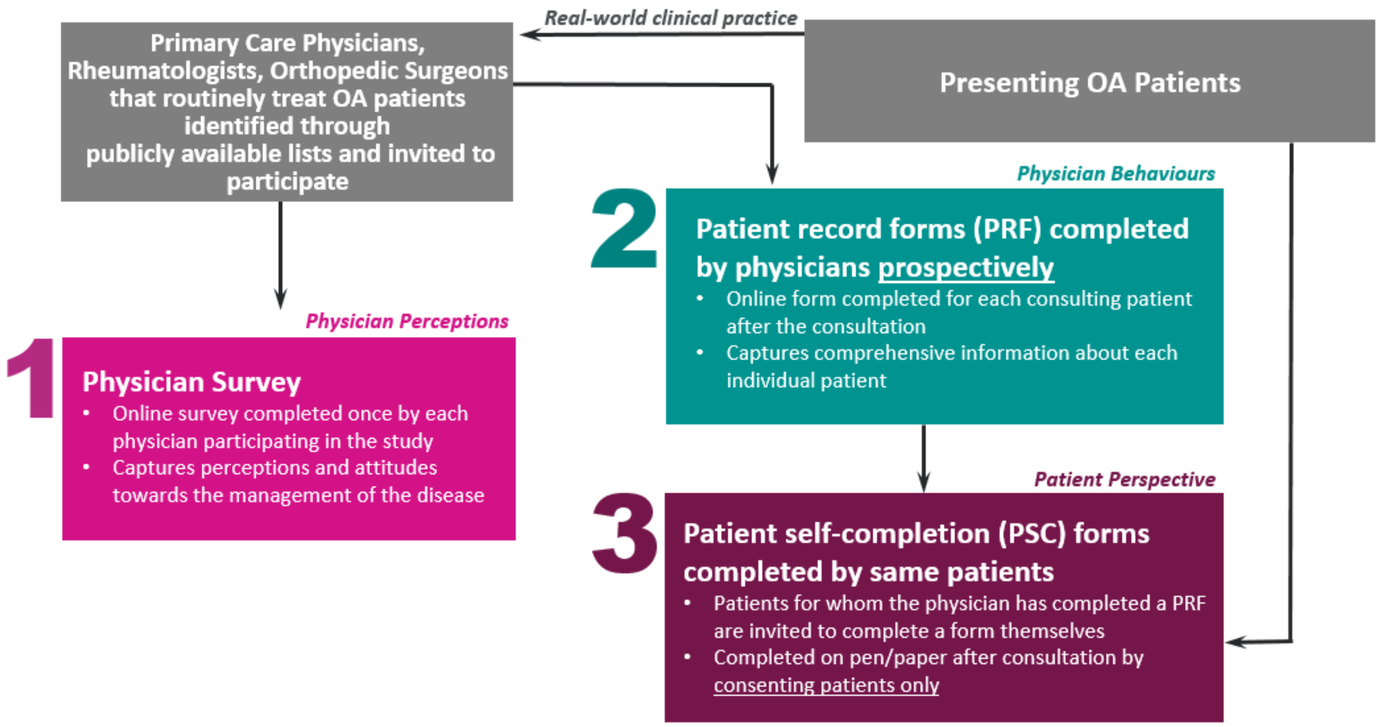

Supplement: Supplementary file 1 [file jcm-12-02733-s001.zip › jcm-2228205-supplementary.pdf]
